# Exploring vitamin D levels and the impact of vitamin D supplementation in pregnant women with diabetes: meta-analysis

**DOI:** 10.1186/s41043-026-01316-8

**Published:** 2026-04-21

**Authors:** Batool Heydarisadegh, Mehrdad Badkoobeh Hezaveh, Mahboubeh Saljoughi, Mohammad Javad Sanjari, Alireza Amirabadizadeh

**Affiliations:** 1 Emergency Medicine, Department of Emergency Medicine, School of Medicine, Amir al momenin Hospital, Zabol University of Medical Sciences, Zabol, Iran; 2 Division of public Health, Sports & Wellbeing, University of Chester, England, UK; 3 Department of psychology, Zahedan Islamic Azad University, Zahedan, Iran; 4 Islamic azad university of birjand, birjand, Iran; 5 Medical Toxicology and Drug Abuse Research Center (MTDRC), Birjand University of Medical Sciences, Birjand, Iran

**Keywords:** Vitamin D, Pregnant Women, Diabetes, Meta-analysis, Gestational diabetes mellitus

## Abstract

Gestational diabetes mellitus (GDM) poses substantial risks to maternal and neonatal health, with a rising global prevalence. Emerging evidence suggests that vitamin D deficiency may contribute to the development of GDM. This meta-analysis aimed to evaluate the association between serum vitamin D levels and GDM, as well as the effects of vitamin D supplementation in pregnant women with diabetes.

A comprehensive systematic search of the Web of Science, PubMed, Scopus, and Cochrane databases was conducted through December 2024. Randomized controlled trials and observational studies involving pregnant women with GDM were included. Vitamin D supplementation was initiated during pregnancy, primarily in the second trimester, with dosages ranging from 400 IU/day to 50,000 IU/week. Risk of bias was assessed using the Cochrane Collaboration tool.

Of the 2,250 identified articles, 68 studies met the inclusion criteria. A pooled analysis demonstrated significantly lower serum vitamin D levels in women with GDM compared with controls (Hedges’ g = − 0.45; 95% CI: −0.65 to − 0.25). In intervention studies, vitamin D supplementation significantly increased serum vitamin D levels compared with placebo (Hedges’ g = 1.38; 95% CI: 0.72 to 2.03), with a similarly significant improvement observed when comparing pre- and post-intervention levels (Hedges’ g = 1.74; 95% CI: 1.06 to 2.41).

Vitamin D supplementation appears effective in correcting vitamin D deficiency among pregnant women with GDM and may improve metabolic outcomes. Further research is warranted to determine optimal dosing regimens, timing of supplementation during pregnancy, and the underlying biological mechanisms linking vitamin D status to glucose metabolism and insulin resistance in GDM.

**Supplementary Information:**

The online version contains supplementary material available at 10.1186/s41043-026-01316-8.

## Introduction

Gestational diabetes mellitus (GDM) is a pregnancy-specific metabolic disorder characterized by glucose intolerance resulting from insulin resistance and/or impaired pancreatic β-cell function [1, 2]. It represents one of the most common complications of pregnancy and is associated with adverse short- and long-term outcomes for both mothers and offspring [3–5]. Globally, approximately one in ten pregnancies is affected by diabetes, with GDM accounting for the majority of cases [6, 7]. In Iran, the prevalence of GDM is reported to range between 5% and 10% [8].

If left undiagnosed or inadequately managed, GDM increases the risk of maternal complications such as preeclampsia, cesarean delivery, and future type 2 diabetes, as well as neonatal complications including macrosomia, hypoglycemia, and respiratory distress [9–11]. Given the high global prevalence of GDM, reaching 15.08%, preventing and treating GDM is crucial for pregnant women and their infants [5].

Vitamin D is a fat-soluble micronutrient that plays a critical role not only in calcium homeostasis and bone metabolism but also in glucose regulation and insulin sensitivity [12, 13]. Vitamin D receptors are widely expressed in pancreatic β-cells, adipose tissue, and skeletal muscle, suggesting a potential role in insulin secretion and glucose metabolism [14, 15]. Experimental and clinical evidence indicate that vitamin D may enhance insulin secretion, improve insulin sensitivity, and modulate inflammatory pathways involved in glucose homeostasis [1, 14, 16, 17].

Vitamin D deficiency is highly prevalent among pregnant women worldwide, with reported rates ranging from 1% to 90%, and particularly high prevalence in the Middle East [1, 14, 18, 19]. Factors such as limited sunlight exposure, inadequate dietary intake, increased adiposity, and increased fetal demand during late pregnancy contribute to reduced serum vitamin D levels [19]. This widespread deficiency has raised concerns regarding its potential contribution to pregnancy-related metabolic disorders, including GDM.

Several observational studies have reported an association between low maternal serum vitamin D levels and an increased risk of GDM [5, 20–22]. Proposed mechanisms include impaired β-cell function, increased insulin resistance mediated by secondary hyperparathyroidism, and dysregulation of inflammatory and oxidative stress pathways [23–25]. However, evidence from interventional studies evaluating vitamin D supplementation during pregnancy has been inconsistent, with some trials demonstrating improvements in vitamin D status and glycemic indices. In contrast, others report minimal or no effect on glucose outcomes [26, 27].

Importantly, many previous studies do not clearly distinguish between observational associations and the potential causal effects of vitamin D supplementation, and results vary according to study design, dosage, timing of supplementation, and baseline vitamin D status. This inconsistency underscores the need for a comprehensive synthesis of both observational and interventional evidence.

Therefore, the present meta-analysis was conducted to systematically evaluate the association between maternal serum vitamin D levels and the risk of gestational diabetes, and the effect of vitamin D supplementation on vitamin D status among pregnant women with GDM. By integrating data from randomized controlled trials and observational studies, this study aims to clarify existing inconsistencies and provide a more robust evidence base for future clinical and preventive strategies.

## Methods

The search was conducted across the Web of Science, PubMed, Scopus, and Cochrane databases through December 2024. The search terms, including gestational diabetes and vitamin D, are detailed in the supplemental literature.

### Eligibility criteria

In the initial phase, eligible articles had to meet the following criteria: (1) randomized and controlled clinical trial design, (2) inclusion of women with gestational diabetes, (3) intervention involving vitamin D supplementation, (4) presence of a control group (administered a placebo), and (5) reporting of vitamin levels in both groups. Trials were excluded if patients required alternative therapies (e.g., insulin, metformin, etc.) or were treated with drugs other than vitamins, minerals, or placebos. Additionally, trials lacking available full-text or post-delivery data were also excluded.

### Literature quality evaluation

Risk of bias for randomized clinical trials was assessed using the Cochrane Collaboration tool, covering sequence generation, allocation concealment, blinding, incomplete data, selective reporting, and other biases, and classified as low, unclear, or high risk. For cross-sectional, case-control, and cohort studies, methodological quality was evaluated using the Newcastle–Ottawa Scale (NOS).

## Data extraction

To extract data from the studies, two independent reviewers conducted screening and extraction. In the event of a conflict between the two reviewers, the study’s author took responsibility for resolving the discrepancy. The extracted information encompassed the author’s name, year of publication, age of the subjects under study, sample size, dosage, duration of follow-up, study type, and vitamin D levels.

### Publication bias assessment

Publication bias was assessed using a funnel plot and Egger’s test, with statistical significance set at *p* < 0.05.

### Statistical analysis

The data analysis was performed using Stata 17 software. Results were presented as mean differences with a 95% confidence interval. To assess heterogeneity between studies, I² and τ^2^ indices were utilized. Statistical heterogeneity was considered present if the I² index exceeded 75% and the p-value was less than 0.05, prompting the use of a random-effects model. Publication bias was evaluated through a funnel plot and Egger regression test. To explore potential sources of heterogeneity, meta-regression analyses were performed based on participants’ mean age and study design. Additionally, sensitivity analyses were conducted to evaluate the robustness of the pooled estimates. A leave-one-out analysis was performed to assess the influence of individual studies on the overall effect size.

## Result

**Fig. 1:**
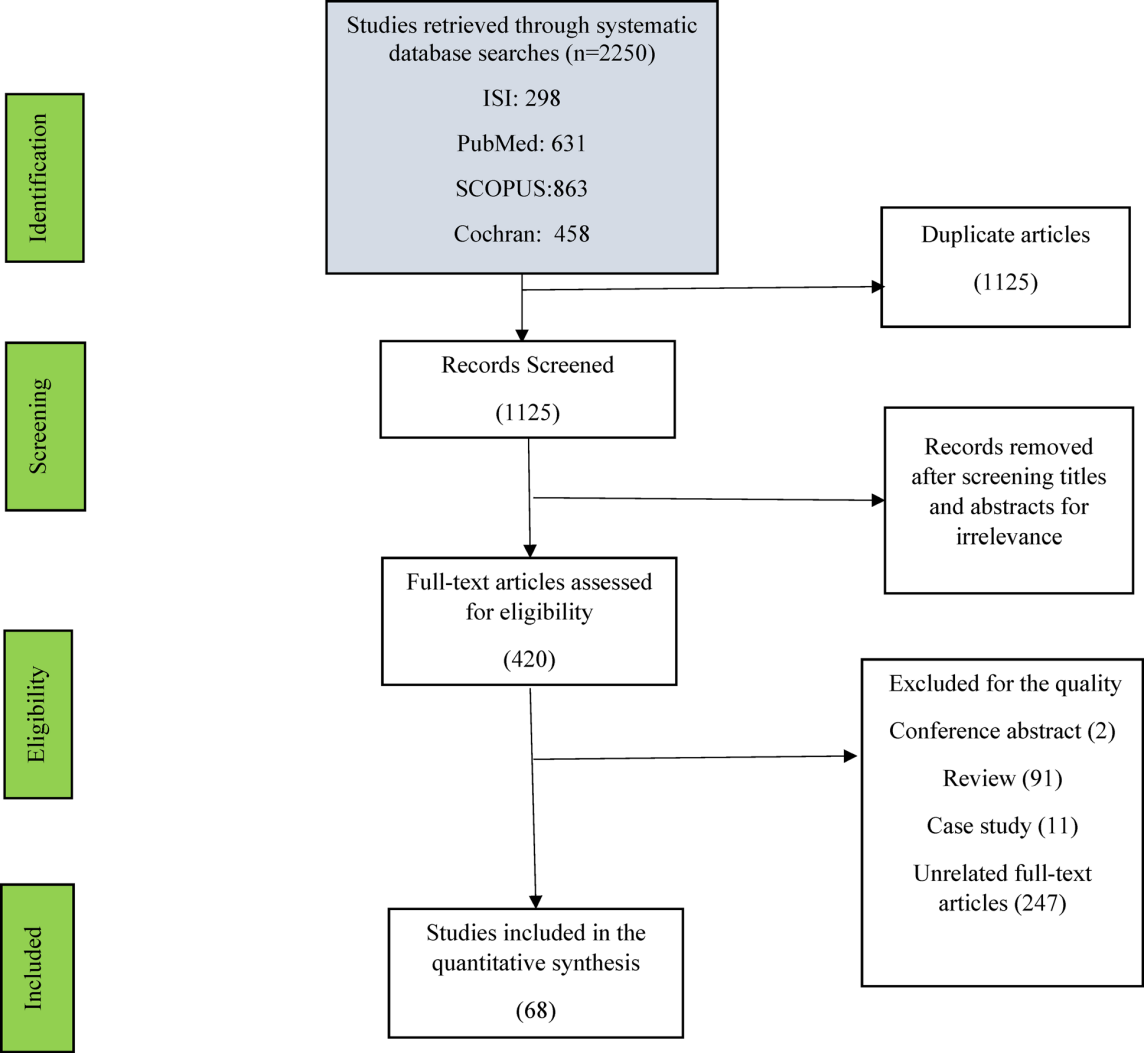
PRISMA Flow Diagram Illustrating Database Search Results and Study Selection

Our initial search of scientific databases yielded 2250 articles. Subsequent screening using EndNote software identified and removed 1125 duplicate articles. Further refinement through title checks led to the exclusion of an additional 705 articles. Ultimately, after a thorough review of abstracts and full-text content, 68 articles met our study criteria. These comprised 15 clinical trials, 16 case-control studies, 5 cohort studies, and 51 descriptive-analytical studies (Fig. 1).

Quality assessment of the clinical trials showed that 10 of 15 studies were judged to have a low risk of bias. Four studies were categorized as having an unclear risk of bias, while one trial was deemed to be at a high risk of bias (Fig S1). The quality assessment of the included studies was conducted using the Newcastle–Ottawa Scale (NOS) for cross-sectional, case-control, and cohort studies. As shown in Tables 1, 2 and 3, all studies met the minimum criteria for inclusion, with NOS scores ranging from 7 to 8 stars, indicating satisfactory methodological quality (Table S1-3).

**Table 1:** Comparing vitamin D levels between the case and control groups in Gestational Diabetes Mellitus

Author (year)	Type study	Sample size intervention group	Sample size control group	Age intervention group	Age control group	Vitamin D intervention group	Vitamin D control group
Prasad das (2023)	cross-sectional	100 people	250 people	25.96 ± 5.5	25.17 ± 5.0	19.5 ± 10.7	20.0 ± 10.7
Yong (2022)	cohort	36 people	223 people	31.21 ± 3.62	30.39 ± 4.53	37.65 ± 10.79	32.05 ± 11.3
Tkachuk (2022)	case-control	138 people	180 people	31 ± 2.1	29 ± 3.57	20 ± 5.4	20.5 ± 6.68
Lotfalizadeh(2022)	cross-sectional	41 people	408 people	-----------	-----------	18 ± 27.75	22 ± 29.25
Cheng (2022)	cohort	669 people	7147 people	32.1 ± 3.4	30.4 ± 3.9	19 ± 6.8	19.6 ± 7.5
Agüero-Domenech (2022)	cross-sectional	93 people	793 people	34.2 ± 5.1	31.7 ± 5.7	17.2 ± 8.6	19.8 ± 8.9
Salakos (2021)	case-control	250 people	941 people	32.8 ± 4.6	32.3 ± 5	21.1 ± 10	22.7 ± 10
pham (2021)	cross-sectional	44 people	1453 people	33.8 ± 4.5	32.2 ± 4.9	1.7 ± 1.98	2.8 ± 3.26
Geng (2021)	cross-sectional	50 people	50 people	-----------	-----------	17.46 ± 5.59	21.51 ± 7.14
Jiang (2021)	case-control	50 people	50 people	26.6 ± 3.4	26.84 ± 3.53	49.94 ± 7.94	55.23 ± 12.06
Ismail (2021)	cross-sectional	58 people	20 people	32 ± 4.3	30.79 ± 4.6	14.43 ± 5.27	15.45 ± 5.29
Bojnordi (2021)	case-control	143 people	470 people	32.62 ± 4.7	30.82 ± 4.61	20.98 ± 16.04	20.99 ± 15.67
Shabrawy (2021)	case-control	52 people	52 people	27.4 ± 4.5	28.3 ± 4.9	15.9 ± 3.8	29.9 ± 5.6
Domenech (2021)	cross-sectional	93 people	793 people	34.2 ± 5.4	31.7 ± 5.7	17.2 ± 8.6	19.5 ± 8.9
Yaqiong (2020)	cross-sectional	110 people	100 people	30.44 ± 3.65	29.68 ± 3.93	13.9 ± 4.88	17.5 ± 5.13
shao (2020)	cross-sectional	718 people	2600 people	29.8 ± 3.8	28.4 ± 3.6	25.9 ± 11.9	26.8 ± 12.2
Ren (2020)	cross-sectional	51 people	48 people	28.54 ± 6.1	28.21 ± 6.31	20.34 ± 5.13	24.52 ± 5.42
RANGARAJU (2020)	cross-sectional	50 people	50 people	30.2 ± 2.8	29.2 ± 2.54	21.08 ± 3.33	30.3 ± 4.04
Collantes-Gutiérrez (2020)	case-control	25 people	25 people	32.32 ± 5.2	30.92 ± 5.06	23.29 ± 9	26.76 ± 9.33
Cabrera (2020)	cross-sectional	56 people	155 people	33.2 ± 5.9	28.7 ± 5.2	21 ± 8.1	18.7 ± 5.3
Zhu (2019)	cohort	399 people	2711 people	28.1 ± 3.8	26.5 ± 3.5	19.8 ± 8.3	18 ± 8.4
Saleem (2019)	case-control	100 people	200 people	-----------	-----------	32.07 ± 22.64	44.76 ± 28.04
Rajput (2019)	case-control	50 people	50 people	25.94 ± 4.9	23.28 ± 4.77	24.7 ± 17.6	45.8 ± 28
Nadimibarforoushi (2019)	case-control	30 people	30 people			11.7 ± 1.7	11.2 ± 0.7
Ede (2019)	case-control	40 people	40 people	32.1 ± 4.88	28.7 ± 4.87	16.9 ± 1.57	21 ± 1.29
Dwarkanath (2019)	cohort	40 people	352 people	25.7 ± 4	23.7 ± 3.7	34 ± 17.4	37.5 ± 19.2
Azzam (2019)	cross-sectional	40 people	40 people			14.01 ± 7.01	16.14 ± 8.57

*: Mean ± SD

### Comparing the effect of vitamin D with the control group in pregnant diabetics

In this section, studies were reviewed across three types: cross-sectional, case-control, and cohort.

In cross-sectional studies, 3589 cases and 16,076 controls were evaluated. In case-control studies, 2799 cases and 6600 controls were included. Cohort studies assessed 1165 cases and 10,448 controls. The mean age of participants in both groups, stratified by study design, is reported in Table 1.

**Fig. 2:**
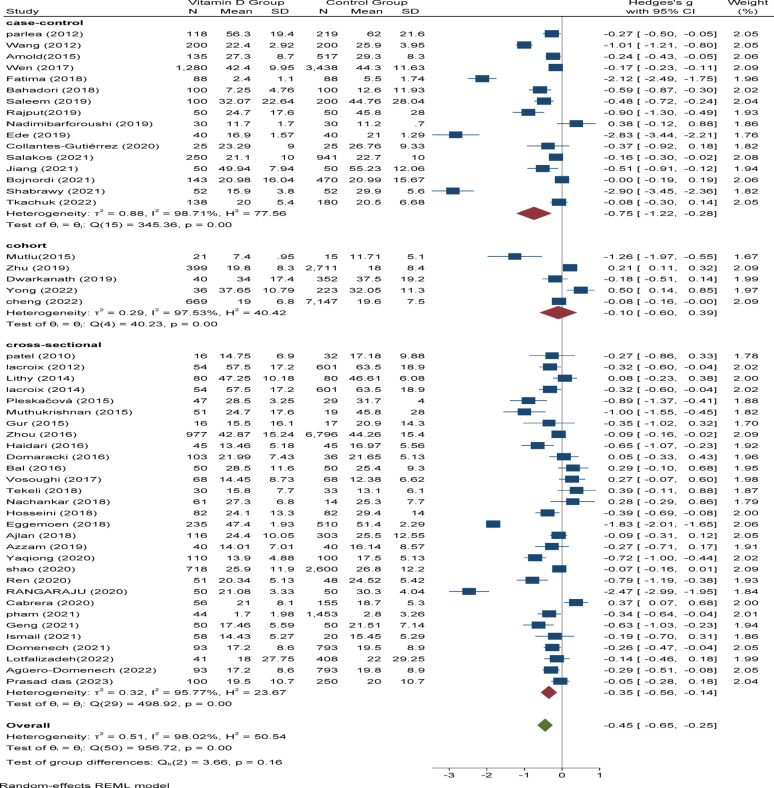
Forest plot comparing vitamin D levels between the case and control groups in Gestational Diabetes Mellitus

The analyzed data exhibited statistical heterogeneity, indicating variability within the study outcomes (I^2^: 98.02, *p* < 0.001). In all three types of studies, the combined results revealed a statistically significant lower level of vitamin D in the case group compared to the control group (Hedges’ g = − 0.45; 95% CI: −0.65 to − 0.25) (Fig. 2).

**Fig. 3:**
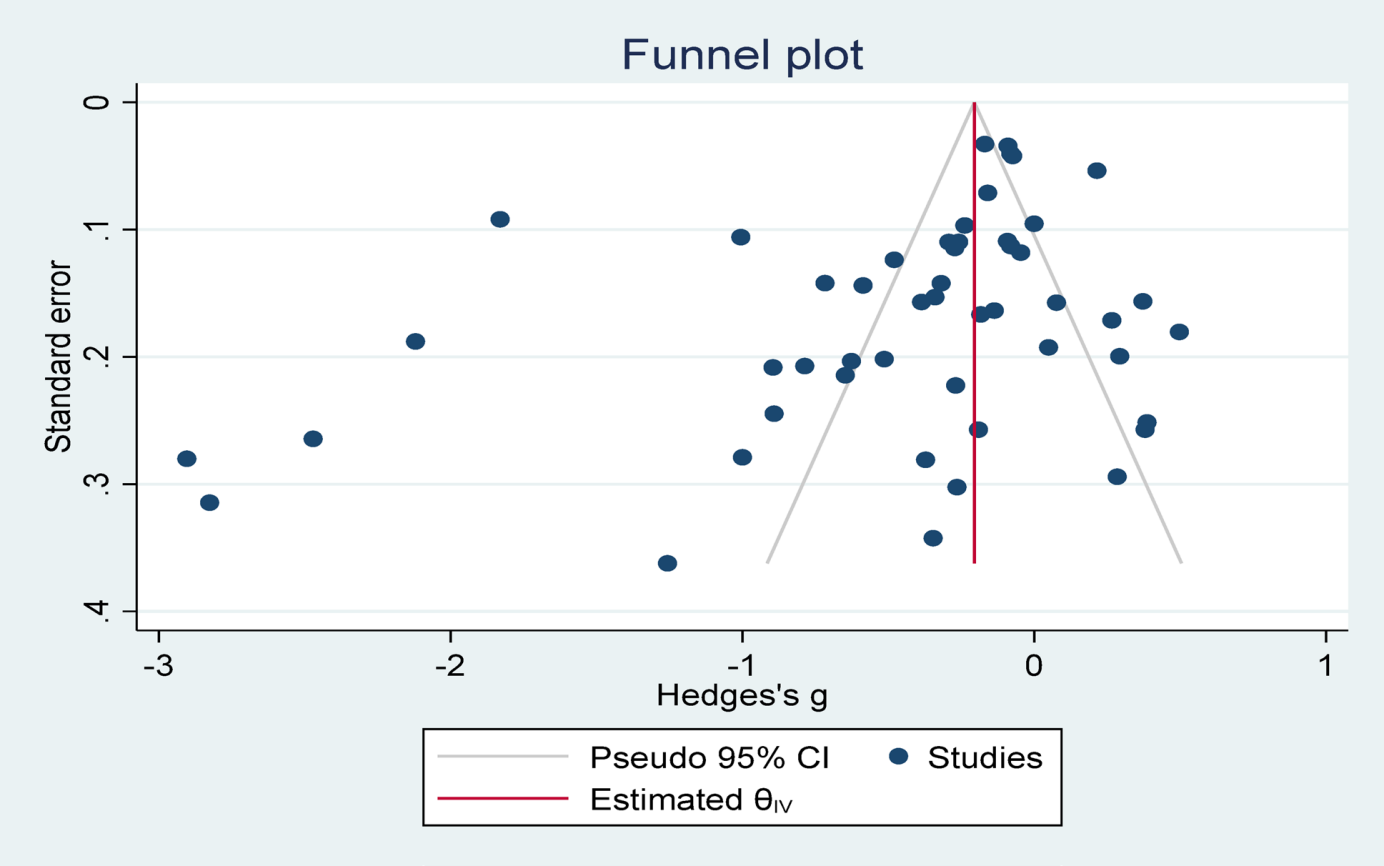
Funnel plots of Hedges’ g effect sizes for the GDM case and control groups

The funnel plot (Fig. 3) and the results of the Egger test (z=-2.51, *p* = 0.01) indicated the presence of publication bias. A leave-one-out sensitivity analysis was conducted to assess the influence of each study on the overall pooled effect size. The sequential omission of single studies did not materially alter the magnitude or direction of the pooled effect estimate, and the results remained statistically significant throughout.

Additionally, meta-regression analyses were performed to explore potential sources of heterogeneity. The results demonstrated that neither participant’s mean age nor study design (cross-sectional, case-control, or cohort) had a statistically significant effect on the pooled effect size (*p* > 0.05), indicating that these variables did not significantly contribute to the observed between-study heterogeneity.

### The impact of vitamin D intervention on patients with gestational diabetes

Fifteen randomized clinical trials (538 in the intervention group and 459 in the placebo group) evaluated the effect of supplementation. The average age in the intervention group was 30.51 years, and in the control group, it was 30.85 years (Table 2).

**Table 2:** comparing vitamin D levels before and after intervention Gestational Diabetes Mellitus

Author (year)	Type Study	Sample size	Age (years)	Vitamin D after intervention	Vitamin D before intervention
Nadeem (2023)	RCT	17 people	25.24 ± 3.2 *	33.9 ± 6.2	17.0 ± 4.8
Azandaryani (2022)	RCT	44 people	25.63 ± 6.08	32.17 ± 13.2	26.51 ± 9.99
Mohammadi (2021)	RCT	15 people	33.3 ± 5.8	30.2 ± 7.4	20.9 ± ± 8.3
Corcoy (2020)	RCT	79 people	32.2 ± 5.2	119.4 ± 35.5	81.9 ± 39.4
Asemi (2020)	RCT	27 people	31.7 ± 5.6	38.95 ± 24.72	20.92 ± 13.97
Jamilian (2019)	RCT	30 people	27.7 ± 4	18.7 ± 4.7	12.6 ± 4.2
Karamali (2018)	RCT	30 people	30 ± 4.5	32.44 ± 16.72	20.71 ± 11.23
Liu (2017)	RCT	84 people	33.3 ± 3.8	74.35 ± 26.13	60.45 ± 23.63
Jamilian (2017)	RCT	35 people	31.5 ± 7	34.4 ± 6.1	16.5 ± 2.6
Yazdchi (2016)	RCT	36 people	31.64 ± 4.4	2.4 ± 0.75	1.3 ± 0.54
Shahgheibi (2016)	RCT	46 people	31.28 ± 6.38	13.5 ± 7.6	17.4 ± 14.9
Karamali (2016)	RCT	30 people	28.7 ± 6.1	36.3 ± 21.3	21.3 ± 14.4
Yeow (2015)	RCT	13 people	36 ± 1	92.4 ± 11.9	28.5 ± 11
Asemi (2014)	RCT	28 people	28.7 ± 6	91.3 ± 54.6	50.8 ± 35.48
shamsi-anr (2012)	RCT	24 people	30.7 ± 6.2	62.1 ± 61.8	24.1 ± ± 43.42

*: Mean ± SD; RCT: Randomized Controlled Trial

**Fig. 4:**
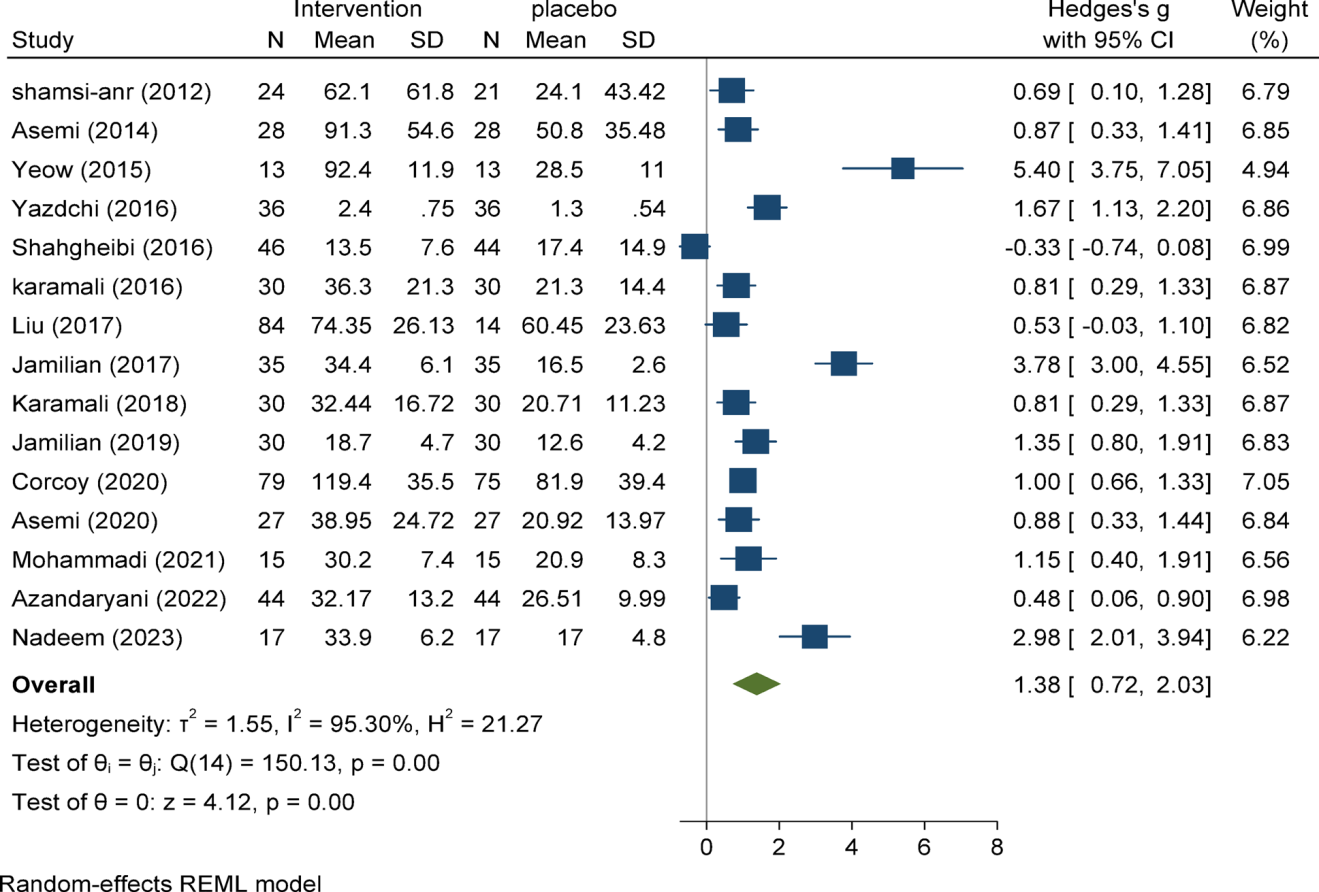
Forest plot comparing vitamin D levels between the intervention and placebo groups in Gestational Diabetes Mellitus

The analyzed data exhibited statistical heterogeneity, indicating variability within the study outcomes (I^2^: 95.30, *p* < 0.001). The aggregated findings revealed a statistically significant increase in vitamin D levels within the intervention group compared to the control group (Hedges’ g = 1.38; 95% CI: 0.72 to 2.03) (Fig. 4).

**Fig. 5:**
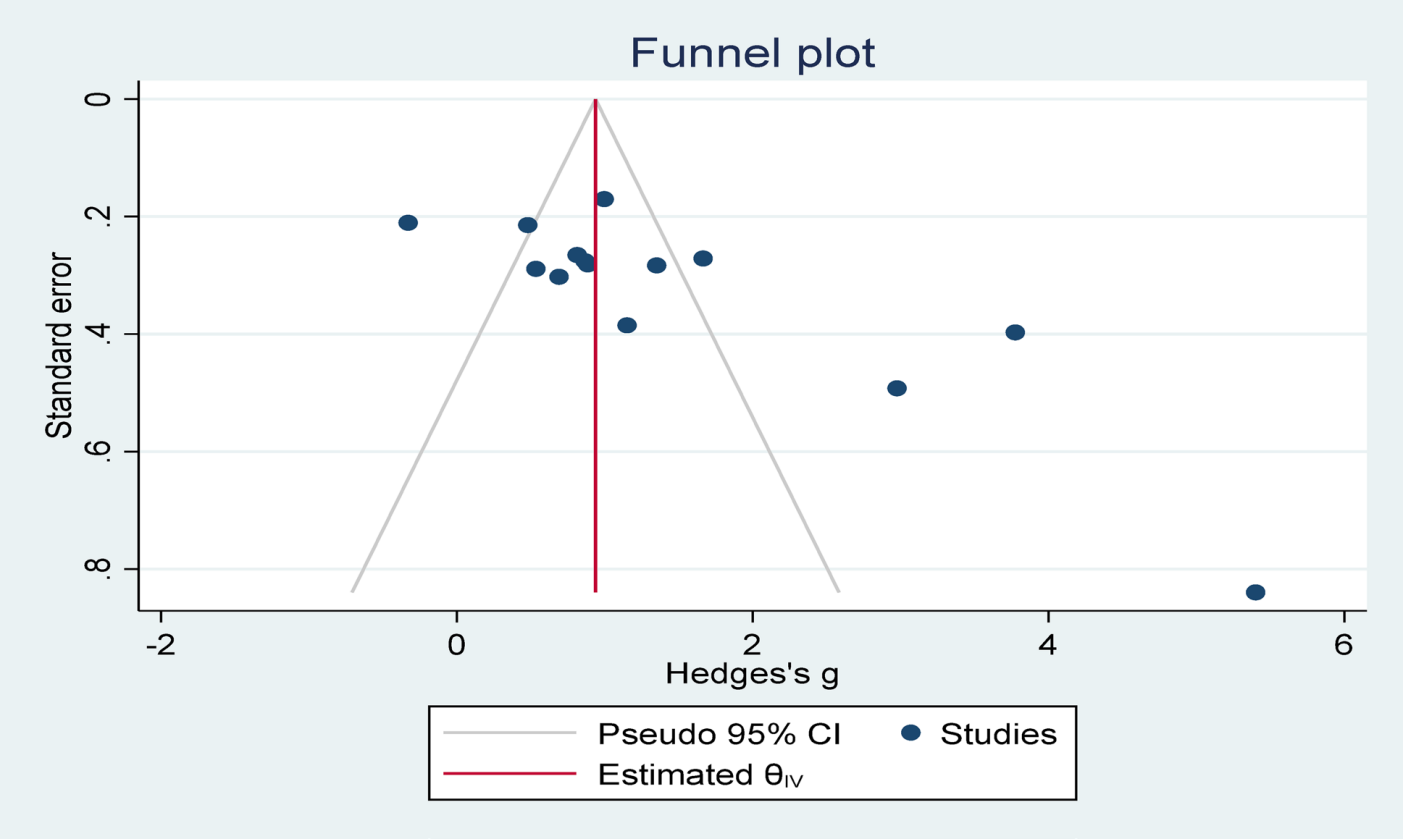
Funnel plots of Hedges’ g effect sizes for the intervention and placebo groups in patients with GDM

The funnel plot (Fig. 5) and the results of the Egger test (z = 5.56, *p* < 0.001) indicated the presence of publication bias. A leave-one-out sensitivity analysis showed that removing individual studies did not materially change the pooled effect size or its statistical significance. These findings confirm the robustness of the overall results.

Meta-regression analysis was conducted to examine whether participants’ mean age influenced the pooled effect size. The results indicated that mean age was not a statistically significant moderator of the intervention effect (*p* > 0.05), suggesting that age did not significantly contribute to the observed between-study heterogeneity.

### Comparing the effect of vitamin D before and after intervention in gestational diabetes patients

In this section, we assessed 14 studies involving 408 individuals. The average age of the subjects included in the studies was 30.23 ± 5.01 years (Table 2).

**Fig. 6:**
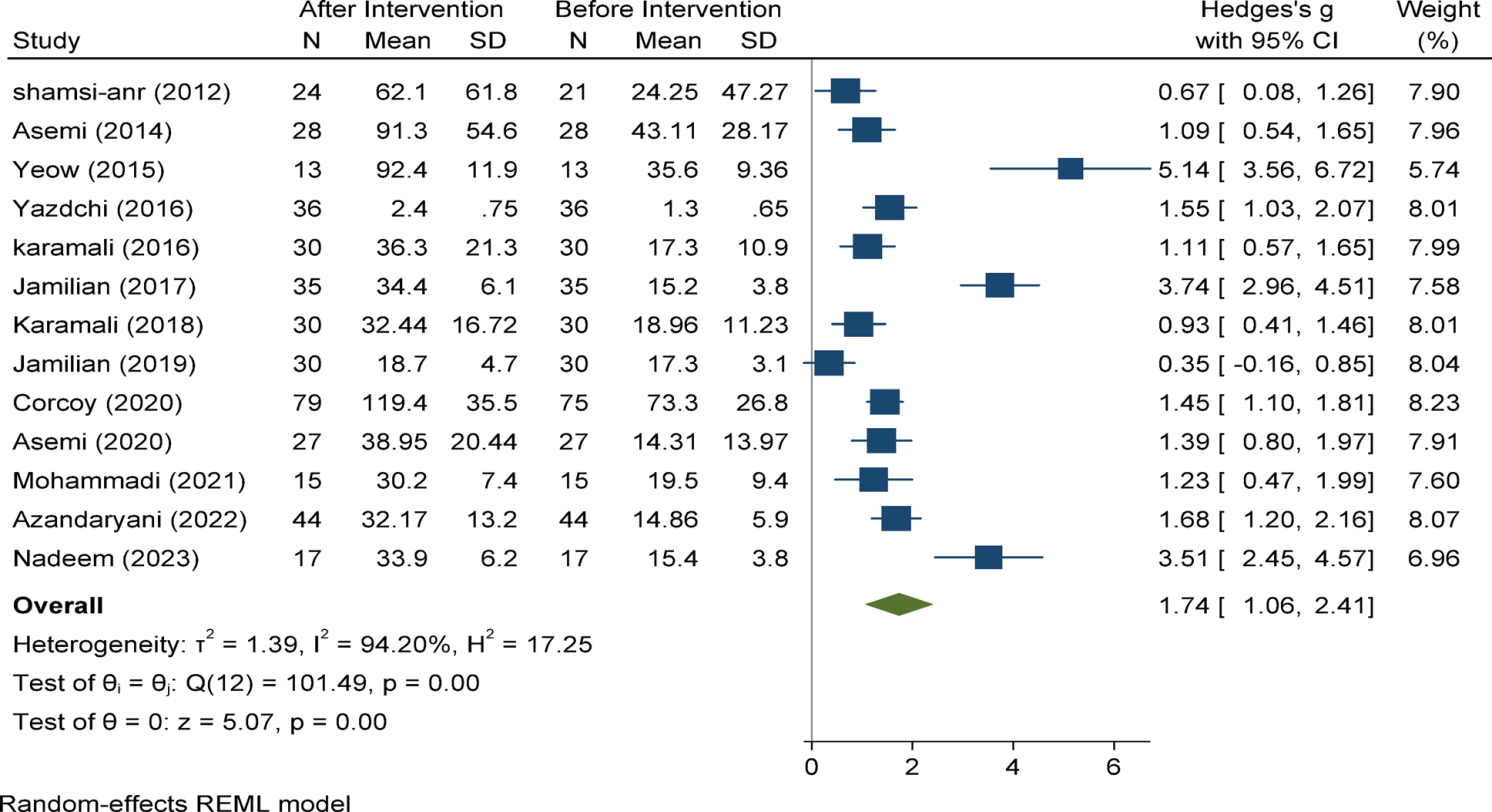
Forest plot comparing vitamin D levels before and after intervention in Gestational Diabetes Mellitus

Significant heterogeneity was observed among the studies included in the analysis, indicating notable variations in the data (I^2^: 94.20, *p* < 0.001). The pooled results indicated a significant increase in average vitamin D levels after the intervention compared to before (Hedges’ g = 1.74; 95% CI: 1.06 to 2.41) (Fig. 6).

**Fig. 7:**
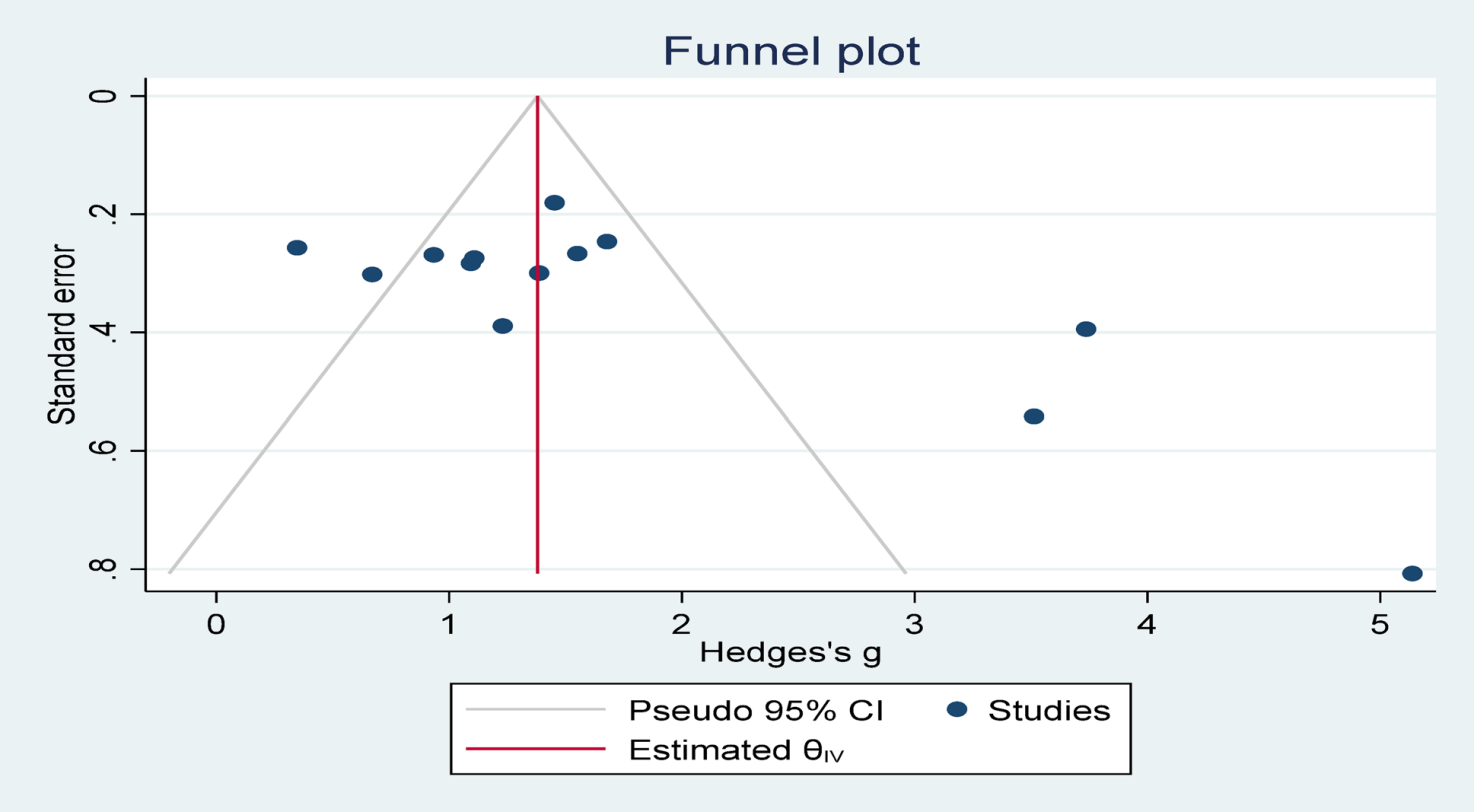
Funnel plots of Hedges’ g effect sizes for pre- and post-intervention comparisons in patients with GDM

The funnel plot (Fig. 7) and the results of the Egger test (z = 4.48, *p* < 0.001) indicated the presence of publication bias. A leave-one-out sensitivity analysis showed that removing individual studies did not materially change the pooled effect size or its statistical significance. These findings confirm the robustness of the overall results.

Meta-regression analysis showed that participants’ mean age did not significantly influence the pooled effect size (*p* > 0.05). Therefore, age was not identified as a significant source of between-study heterogeneity.

## Discussion

Vitamin D deficiency remains highly prevalent among pregnant women worldwide, ranging from 40% to 100% across different populations [18, 28, 29]. Our meta-analysis confirms that pregnant women with GDM generally have lower serum vitamin D levels than controls. Vitamin D plays a key role in insulin synthesis, secretion, and sensitivity, influencing glucose metabolism and the development of GDM [5, 30]. 

The pathogenesis of GDM is not precisely determined, but previous studies have suggested genetic differences, insulin resistance, damage to beta pancreatic cells, and immune system dysfunction. Insulin sensitivity correlates directly with serum 25-hydroxyvitamin D levels, and vitamin D deficiency impairs the function of beta pancreatic cells [31]. Dysfunction in beta cells leads to reduced insulin production and the onset of gestational diabetes [5, 31–38]. Johns and colleagues stated in their study that GDM involves the inadequacy of beta pancreatic cells in responding adequately to the increased insulin requirements during pregnancy, resulting in varying degrees of hyperglycemia. The pathophysiological features of insulin resistance and impaired insulin secretion reflect observations in type 2 diabetes mellitus (T2DM) [39]. Overall, vitamin D plays a crucial role in maintaining normal glucose levels and reduces the associated damage with insulin resistance [40]. Adequate vitamin D is necessary for the natural production and secretion of insulin by the pancreatic islets [35]. 

Factors affecting serum vitamin D, such as seasonal sunlight exposure, outdoor activity, and supplementation, were considered, but they are summarized here to avoid overemphasis [41, 42]. Our results indicate that vitamin D supplementation significantly increases serum levels, demonstrating intervention efficacy, though evidence for reducing GDM incidence or improving maternal/neonatal outcomes remains less conclusive. Observational associations cannot establish causality, and findings should be interpreted accordingly. Results from some studies suggest that vitamin D supplementation improves maternal vitamin D status during pregnancy [41]. Our findings indicated that the intervention group receiving vitamin D had higher vitamin D levels, demonstrating the intervention’s meaningfulness. Increasing vitamin D intake among mothers can reduce the risk of adverse pregnancy outcomes. Maintaining an adequate vitamin D level in the serum is essential to support fetal growth and reduce health problems for pregnant women.

Limitations of this study include high heterogeneity, variability in vitamin D assays, differences in diagnostic criteria for GDM, potential confounding by BMI, ethnicity, diet, and sun exposure, and publication bias, which may have inflated observed effects. These factors should be considered when applying the results to clinical practice.

Future research should focus on optimal vitamin D dosing, timing of supplementation, and high-quality randomized trials assessing clinically relevant maternal and neonatal outcomes. The findings of this study, while based largely on Iranian and regional populations, may inform international practice by highlighting the potential benefit of monitoring and correcting vitamin D deficiency during pregnancy to improve maternal glucose metabolism and reduce the risk of GDM globally.

## Conclusion

This meta-analysis shows that vitamin D deficiency is common among pregnant women worldwide and is associated with an increased risk of gestational diabetes mellitus (GDM). Women with GDM consistently exhibit lower serum vitamin D levels than controls. Vitamin D supplementation effectively raises circulating levels, supporting insulin synthesis, secretion, and glucose regulation. Although evidence for reducing GDM incidence or improving clinical outcomes is less robust, maintaining adequate vitamin D during pregnancy is a modifiable factor with potential global health benefits. Population-specific factors such as sun exposure, diet, BMI, and ethnicity influence vitamin D status. High-quality randomized trials are needed to define optimal dosing, timing, and maternal–neonatal outcomes.

## Electronic Supplementary Material

Below is the link to the electronic supplementary material.

Supplementary Material 1

## Data Availability

The datasets utilized and/or analyzed during the present study are accessible upon reasonable request from the corresponding author.
