# Supplementary material for: Exploring vitamin D levels and the impact of vitamin D supplementation in pregnant women with diabetes: meta-analysis
Source: J Health Popul Nutr. 2026 Apr 21;45:154. doi: 10.1186/s41043-026-01316-8 (PMC13289124; doi:10.1186/s41043-026-01316-8)
Supplement: Supplementary file 1 — Supplementary Material 1 [file 41043_2026_1316_MOESM1_ESM.docx]

Table S1: Quality Assessment of Cross-Sectional Studies Using the Newcastle–Ottawa Scale (NOS)

| Author (year) | Representativeness | Sample size | Non-respondents | Exposure ascertainment | Outcome measurement | Comparability | Outcome assessment | Total NOS |
| --- | --- | --- | --- | --- | --- | --- | --- | --- |
| Prasad das (2023) | ★ | ★ | ★ | ★ | ★ | ★ | ★ | 7 |
| Lotfalizadeh (2022) | ★ | ★ | ★ | ★ | ★ | ★ | ★ | 7 |
| Agüero-Domenech (2022) | ★ | ★ | ★ | ★ | ★ | ★ | ★ | 7 |
| pham (2021) | ★ | ★ | ★ | ★ | ★ | ★ | ★ | 7 |
| Geng (2021) | ★ | ★ | ★ | ★ | ★ | ★ | ★ | 7 |
| Ismail (2021) | ★ | ★ | ★ | ★ | ★ | ★ | ★ | 7 |
| Domenech (2021) | ★ | ★ | ★ | ★ | ★ | ★ | ★ | 7 |
| Yaqiong (2020) | ★ | ★ | ★ | ★ | ★ | ★ | ★ | 7 |
| shao (2020) | ★ | ★ | ★ | ★ | ★ | ★ | ★ | 7 |
| Ren (2020) | ★ | ★ | ★ | ★ | ★ | ★ | ★ | 7 |
| RANGARAJU (2020) | ★ | ★ | ★ | ★ | ★ | ★ | ★ | 7 |
| Cabrera (2020) | ★ | ★ | ★ | ★ | ★ | ★ | ★ | 7 |
| Azzam (2019) | ★ | ★ | ★ | ★ | ★ | ★ | ★ | 7 |

Tables2: Quality Assessment of Case-Control Studies Using the Newcastle–Ottawa Scale (NOS)

| Author (year) | Case definition | Representativeness | Control selection | Control definition | Comparability | Exposure ascertainment | Same method | Non-response | Total NOS |
| --- | --- | --- | --- | --- | --- | --- | --- | --- | --- |
| Tkachuk (2022) | ★ | ★ | ★ | ★ | ★ | ★ | ★ | ★ | 8 |
| Salakos (2021) | ★ | ★ | ★ | ★ | ★ | ★ | ★ | ★ | 8 |
| Jiang (2021) | ★ | ★ | ★ | ★ | ★ | ★ | ★ | ★ | 8 |
| Bojnordi (2021) | ★ | ★ | ★ | ★ | ★ | ★ | ★ | ★ | 8 |
| Shabrawy (2021) | ★ | ★ | ★ | ★ | ★ | ★ | ★ | ★ | 8 |
| Collantes-Gutiérrez (2020) | ★ | ★ | ★ | ★ | ★ | ★ | ★ | ★ | 8 |
| Saleem (2019) | ★ | ★ | ★ | ★ | ★ | ★ | ★ | ★ | 8 |
| Rajput (2019) | ★ | ★ | ★ | ★ | ★ | ★ | ★ | ★ | 8 |
| Nadimibarforoushi (2019) | ★ | ★ | ★ | ★ | ★ | ★ | ★ | ★ | 8 |
| Ede (2019) | ★ | ★ | ★ | ★ | ★ | ★ | ★ | ★ | 8 |

Table S3: Quality Assessment of Cohort Studies Using the Newcastle–Ottawa Scale (NOS)

| Author (year) | Representativeness | Non-exposed selection | Exposure ascertainment | Outcome at baseline | Comparability | Outcome assessment | Follow-up duration | Completeness | Total NOS |
| --- | --- | --- | --- | --- | --- | --- | --- | --- | --- |
| Yong (2022) | ★ | ★ | ★ | ★ | ★ | ★ | ★ | ★ | 8 |
| cheng (2022) | ★ | ★ | ★ | ★ | ★ | ★ | ★ | ★ | 8 |
| Zhu (2019) | ★ | ★ | ★ | ★ | ★ | ★ | ★ | ★ | 8 |
| Dwarkanath (2019) | ★ | ★ | ★ | ★ | ★ | ★ | ★ | ★ | 8 |


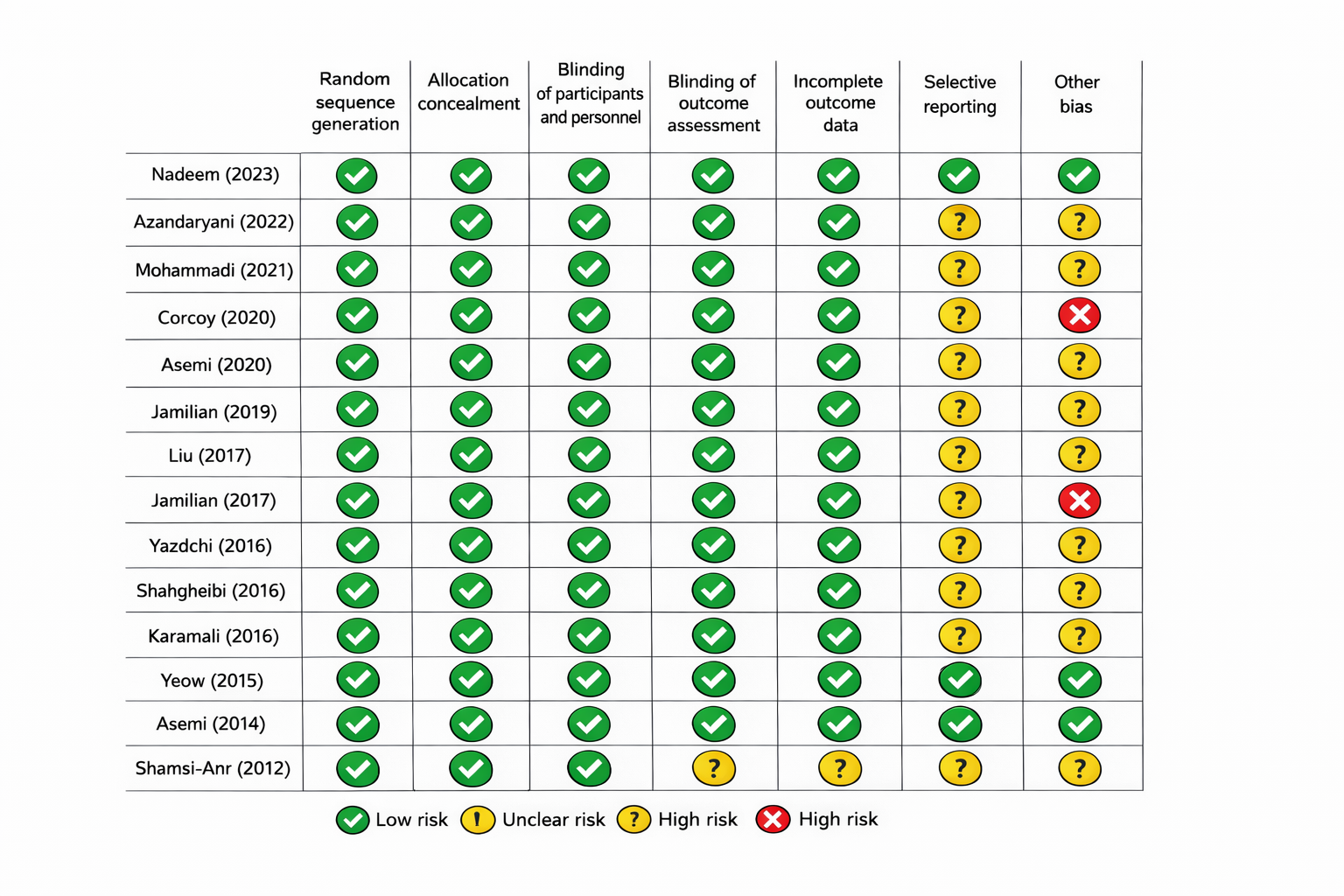


Figure S 1: Risk of Bias Assessment of Included Randomized Controlled Trials Using the Cochrane Collaboration Tool.
